# The Effect of Prognostic Nutritional Index on Infection in Acute Ischemic Stroke Patients

**DOI:** 10.3390/medicina59040679

**Published:** 2023-03-29

**Authors:** Sebnem Nergiz, Unal Ozturk

**Affiliations:** 1 Department of Dietetics and Nutrition, Ataturk Faculty of Health Science, Dicle University, Diyarbakir 21280, Turkey; 2 Department of Neurology, Health Sciences University of Turkey, Diyarbakır Gazi Yasargil Education and Research Hospital, Diyarbakir 21070, Turkey

**Keywords:** nutrition, stroke, infection

## Abstract

*Objectives*: Malnutrition is frequently seen in stroke patients. Malnutrition worsens the prognosis and increases the mortality rate in acute ischemic stroke patients. Malnutrition is a significant factor not only in the initiation of infection but also in its progression. The prognostic nutritional index (PNI) is a new index that evaluates the nutrition and inflammatory status. This study aims to investigate the relationship between PNI and stroke-related infection (SRI) development during hospitalization in patients with acute ischemic stroke. *Materials and Methods*: Acute ischemic stroke was the primary diagnosis for 158 patients who were admitted to the neurology intensive care unit. Patients’ demographic, clinical, and laboratory parameters were recorded. PNI was calculated according to the formula given below. PNI: 10 × serum albumin (g/dL) + 0.005 × total lymphocyte count (mm^3^). PNI > 380 normal, PNI: 350–380 moderate malnutrition risk, PNI < 350 severe malnutrition risk. *Results*: A total of 158 patients with acute ischemic stroke were included in the study. There were 70 male and 88 female patients, whereas the mean age of the patients was 67.79 ± 14.0 years. Nosocomial infection developed in 34 (21%) of the patients. Compared to high PNI scores, patients with low PNI scores were generally older, and the National Institutes of Health Stroke Scale (NIHSS) score, atrial fibrillation, infection, mortality rate, and hospitalization rates were all significantly higher. *Conclusions*: In this study, we discovered that patients with poor PNI had a considerably increased rate of infection development. It is vital to evaluate the nutritional status of patients with acute ischemic stroke during hospitalization.

## 1. Introduction

Cerebrovascular diseases affect the central nervous system. In total, 70% of strokes are caused by Ischemic strokes [1]. Both the morbidity and mortality rates from strokes are high. The development of severe complications in stroke patients is a significant cause of morbidity and morbidity. Malnutrition is often present in stroke patients. Malnutrition is a condition which is often overlooked in hospitalized patients. Malnutrition worsens the prognosis and increases the mortality rate [2]. Malnutrition causes deterioration in the immune system [3]. As a result, resistance to pathogenic microorganisms decreases, which results in an increase in treatment resistance. This increases the risk of developing an infection in stroke patients [4]. Malnutrition has a significant role not only at the beginning of the infection but also in its advancement. In addition, the immunosuppressive effect due to cerebral injury facilitates the development of such infections [5]. Stroke-related infections are common and serious complications. The incidence of SRI is between 5 and 65%. Infectious complications are lung infections, urinary system infections, and infections of other systems. Therefore, early diagnosis and treatment of SRI are very important [5]. Effective assessment techniques are crucial for the prevention of SRI in the early period. The clinical nutrition guidelines in neurology advise to assess the nutritional situation of patients during hospitalizations in the early period [6]. Malnutrition significantly increases the mortality rate in coronary artery disease and cancers [7,8]. Nutrition can be a reliable indicator to predict the development of infection. Nutritional indicators can be used in the early risk evaluation of SRIs. The prognostic nutritional index (PNI) is a new index which evaluates the nutritional and inflammatory status. PNI evaluates the immunological nutritional status. PNI is calculated from lymphocyte count and serum albumin level [9]. The purpose of this investigation is to research the relationship between PNI and SRI development during the hospitalization of patients with acute ischemic stroke (AIS).

## 2. Materials and Methods

### 2.1. Study Design and Population

Our investigation is retrospective research. A total of 158 patients with the diagnosis of AIS were hospitalized in the neurology intensive care unit. The following are investigation inclusion criteria: (1) Patients with acute ischemic stroke diagnostic criteria, (2) patients who were hospitalized within the first 24 h after the onset of AIS and the diagnosis of a stroke by CT or MRI, (3) patients over the age of 18, (4) only patients whose blood and biochemical parameters were fully studied. The following are study exclusion criteria: absence of whole blood and biochemical parameters, presence of hematological disease in patients, malignancy, previous cerebrovascular accident, pre-stroke infection, and presence of end-stage renal failure and hepatic failure. Laboratory parameters of the patients during their hospitalization were recorded. This research was accepted by the ethics committee of Health Sciences University of Turkey, Gazi Yaşargil Education and Research Hospital with the number 185-2022.

### 2.2. Clinical Evaluation

The cases’ demographic parameters were recorded. The National Institutes of Health Stroke Scale (NIHSS), imaging characteristics, and length of hospital stay were assessed, together with clinical parameters such as hyperlipidemia, hypertension, diabetes, smoking, atrial fibrillation, and prior cerebrovascular disease.

### 2.3. Laboratory Evaluation

Laboratory indicators obtained during hospitalization were evaluated within the first 24 h of the AIS’s onset. Whole blood, biochemistry, troponin, and lipid parameters were registered from the laboratory indicators. PNI was computed for this with the formula given below. PNI: 10 × serum albumin (g/L) + 0.005 × total lymphocyte count (mm^3^). PNI > 380 normal, PNI: 350–380 moderate malnutrition risk, PNI < 350 severe malnutrition risk [3].

### 2.4. Diagnosis of Infection Associated with Acute Ischemic Stroke

SRI was defined as an infection diagnosed during the hospitalization. The diagnosis of the infection was made by a competent clinician using laboratory and clinical findings according to “The USA Center for Disease Control Recommendation” [10]. SAI was divided into three groups: pneumonia, urinary tract infections, and other infections.

### 2.5. Statistical Analysis

Statistical analysis was performed using the SPSS 14.0 statistical program. All demographic, clinical, and laboratory data were analyzed. The normal distribution of the data was evaluated by utilizing the Kolmogorov–Smirnow test. The Mann–Whitney U test was performed to evaluate the non-normally distributed continuous parameters and the Independent Samples T-test was used in the analysis of normally distributed continuous data. Continuous parameters were stated as mean ± SD. Categorical data were expressed as a percentage and an absolute frequency. To compare categorical data, the chi-square test was used. The Spearman test was used to analyze correlation analyses. Linear and logistic regression analyzes were performed. A value of *p* < 0.05 was considered statistically significant.

## 3. Results

In the investigation, 158 cases of AIS were registered in total. There were 88 female and 70 male patients. The mean age of the patients was 67.79 ± 14.0 years. SAI was observed in 34 (21%) of the patients. The most common infection was urinary tract infection (16 patients, 47%). There were 10 patients with pneumonia who constituted 29% of the total. Other infections were seen in eight (23%) of the patients. The most common agents were *Staphylococcus aureus* (29%), *Escherichia coli ESBL +* (23%), *Staphylococcus haemolyticus* (6%), *Pseudomonas spp.* (12%), *Klebsiella pneumoniae ESBL* + (13%), *Enterobacter cloacae* (6%), *Enterococcus faecium* (5%), *Staphylococcus epidermidis *(6%). The in-hospital mortality rate of the patients was 18%. The mean hospital stay of the patients were 8.3 ± 7.11 days. For the PNI value, the cases were divided into two groups. Patients with a PNI >380 were defined as patients without malnutrition, whereas patients with a PNI ≤ 380 were defined as patients with malnutrition. Compared to high PNI scores, patients with low PNI scores were older, and their NIHSS score, atrial fibrillation, infection, mortality rate, and hospitalization rate were significantly higher (Table 1). In patients with poor PNI, it was discovered that the values for urea, troponin and triglyceride values were much greater. Cholesterol and total protein values were significantly lower in patients with low PNI (Table 2).

### 3.1. Correlation Analysis

PNI was negatively correlated with age, urea, troponin, hospitalization duration, and mortality. PNI was positively correlated with total protein, and cholesterol parameters (Table 3).

### 3.2. Regression Analysis

A statistically significant correlation between PNI and age, urea, total protein, cholesterol parameters, troponin, and hospital stay was discovered in the linear regression analysis (Table 4). A statistically significant association was detected between PNI and infection, NIHSS, atrial fibrillation, hyperlipidemia, and mortality (Table 5).

## 4. Discussion

Cerebrovascular diseases are the most prominent causes of morbidity and mortality [11]. In recent years, it has been reported that malnutrition leads to an increase in the prevalence of infections in AIS patients [3]. Numerous studies reported that malnutrition affects many hormones, organs, cytokine values, and even the functions of immune cells [12,13]. Malnutrition is related to immunosuppression and increases the susceptibility to infections [14]. In stroke patients with malnutrition, early nutritional therapy may improve the outcome of patients.

In our research, we investigated the relationship between PNI at the time of hospitalization and the development of infection in patients with AIS. In this clinical investigation, we discovered a significant link between PNI and infection during admission to the hospital. In patients with AIS, the risk of infection was determined to be significantly greater in cases with PNI ≤ 380 (*p* <0.005). In total, 31% of AIS patients had a normal nutritional status. However, 69% of AIS patients were determined to be malnourished. It was discovered that the infection rate was 21% in acute ischemic stroke patients. Infection adversely affects the prognosis of strokes with different methods. Rehabilitation may be delayed by immobility and poor health in general brought on by prolonged hospitalization. In clinical studies, it has been shown that nutrition is linked to both pneumonia and urinary system infection [15]. The frequent reason for infection in AIS patients was the urinary system infections. The second most frequent cause of infection in AIS patients was pneumonia. It has been reported that the development of infection is related to an increased in-hospital mortality [16]. Malnutrition during hospitalization in AIS patients was associated with poor prognosis, according to earlier investigations [17]. The association between malnutrition and the development of infection and mortality can be described by factors that constitute PNI [3]. In AIS, albumin has neuroprotective properties. Albumin has a protective role against oxidant agents [3]. Low serum albumin values were linked with poor outcomes in stroke patients, according to Dziedzic et al. [18]. According to Kim et al., patients with AIS who had low lymphocyte values had worse prognosis [19].

The lymphocyte level is an important mediator of cellular immunity. In certain malignancies, lymphocytes are a part of the cellular immunity system in different cancers. Lymphocytes have been linked to the prognosis of cancer. Some clinical researchers have proposed the utilization of lymphocyte level as a prognostic indicator [20]. Previous clinical studies reported that acute ischemic stroke resulted in a drop in lymphocyte count. It has been shown that lymphocyte level is related to a bad outcome in acute ischemic stroke patients [21].

In our study, it was discovered that low PNI was related to high NIHSS and mortality. Patients with low PNI were associated with advanced age, increased incidence of atrial fibrillation, increased risk of infection, prolonged hospitalization periods, and increased in-hospital mortality. High mortality was discovered in AF patients with low PNI by Cai J et al. [19]. According to Shang S et al., peritoneal dialysis patients with low PNI had a significantly increased risk of developing pneumonia [22]. Total cholesterol is an important indicator of dietary intake. Low cholesterol values are associated with malnutrition, increased inflammatory situation, and increased mortality in AIS patients, according to prior research. Low cholesterol levels have been linked to both early and long-term mortality in AIS patients [23]. In this research, cholesterol values were determined to be significantly lower in the patient group with low PNI. Our study suggests that the nutritional status of AIS patients should be taken into account. Malnutrition is often overlooked in clinical practice. However, malnutrition has a significant role in immunosuppression. Detection of malnutrition in the early period can determine the risk status and allow an early treatment. Many indices investigate malnutrition status. However, PNI is an easy and reliable index that investigates malnutrition status in clinical practice.

## 5. Conclusions

In this study, we hypothesized that patients with low PNI had a noticeably increased rate of infection development. During hospitalization, it is important to assess the nutritional situation of AIS patients. Providing nutritional support to patients with poor nutritional status can reduce the morbidity and mortality rates of stroke patients. More investigations are needed to assess the effect of nutritional therapy on stroke patients.

### Study Limitations

Our study is a retrospective and single-center study. The blood parameters of the patients were only checked within the first 24 h after hospitalization.

## Figures and Tables

**Table 1:** Clinical characteristics of patients.

	PNI ≤ 380 (n = 110)	PNI > 380 (n = 48)	*p* Value
Age (year)	71.42 ± 12.39	59.88 ± 14.78	0.000
Gender (F/M)	56/54	32/16	0.082
Hypertension	66 (60%)	32 (66%)	0.479
Diabetes Mellitus	30 (27%)	14 (29%)	0.848
Hyperlipidemia	28 (25%)	20 (41%)	0.059
Ischemic Heart Disease	40 (36%)	14 (29%)	0.467
Atrial fibrillation	26 (23%)	4 (8%)	0.027
Infection	27 (24%)	7 (15%)	0.021
NIHSS	17	9	0.029
Non-survivor patients	23 (20%)	5 (10%)	0.037
Hospitalization day	11.04 ± 10.71	7.05 ± 4.45	0.001

PNI: Prognostic Nutritional Index, F: Female, M: Male, NIHSS: National Institutes of Health Stroke Scale.

**Table 2:** Laboratory parameters of patients.

	PNI ≤ 380 (n = 110)	PNI > 380 (n = 48)	*p* Value
WBC (10^3^/µL)	9.33 ± 3.61	9.71 ± 2.42	0.511
Neutrophil (10^3^/µL)	6.39 ± 3.09	6.40 ± 2.48	0.974
Lymphocyte (10^3^/µL)	2.12 ± 0.93	2.54 ± 1.15	0.017
Monocyte (10^3^/µL)	2.18 ± 1.07	0.55 ± 0.17	0.312
Thrombocyte (10^3^/µL)	280.89 ± 116.42	255.12 ± 54.69	0.146
Hemoglobin (g/dL)	15.39 ± 4.22	13.83 ± 1.81	0.554
Glucose (mg/dL)	140.16 ± 70.21	139.41 ± 60.05	0.949
Urea (mg/dL)	50.38 ± 25.94	40.91 ± 14.43	0.019
Creatinin (mg/dL)	1.14 ± 0.43	0.89 ± 0.35	0.175
eGFR (ml/minute/1.73 m^2^)	70.49 ± 22.04	76.13 ± 17.96	0.129
Total protein (g/L)	67.49 ± 7.49	74.66 ± 6.62	0.000
Albumin (g/L)	32.76 ± 3.93	40.45 ± 2.44	0.000
CRP (mg/L)	3.30 ± 1.19	4.68 ± 1.74	0.341
Troponin (ng/L)	17.09 ± 5.71	0.10 ± 0.04	0.008
Total cholesterol (mg/dL)	167.09 ± 40.76	195.66 ± 30.07	0.000
LDL (mg/dL)	100.78 ± 28.56	118.37 ± 29.28	0.001
HDL (mg/dL)	38.33 ± 9.03	46.33 ± 13.72	0.000
Triglyceride (mg/dL)	318.56 ± 78.93	147.62 ± 56.62	0.000

WBC: White Blood Cell, eGFR: e Glomerular Filtration Rate, LDL: Low-Density Lipoprotein, HDL: High-Density Lipoprotein, CRP:C-Reactive Protein.

**Table 3:** Correlation analysis between PNI and clinical parameters in patients with acute ischemic stroke.

Parameters	Spearman’s Correlation Coefficient (*r*)	*p* Value
Age	−0.361	0.000
Lymphocyte	0.162	0.042
Glucose	−0.063	0.429
Urea	−0.165	0.039
Total protein	0.427	0.000
Albumin	0.997	0.000
Total cholesterol	0.403	0.000
LDL	0.309	0.000
HDL	0.271	0.001
Triglyceride	0.071	0.381
Troponin	−0.470	0.000
Hospitalization day	−0.176	0.027

**Table 4:** Linear regression analysis between PNI and clinical parameters in patients with acute ischemic stroke.

Parameters	Β	R Square	*p* Value
Age	−0.373	0.134	0.000
Hemoglobin	−0.009	0.000	0.912
Urea	−0.264	0.069	0.001
Total protein	0.368	0.135	0.000
Total cholesterol	0.343	0.118	0.000
LDL cholesterol	0.303	0.092	0.000
HDL cholesterol	0.320	0.103	0.000
Troponin	−0.334	0.112	0.000
Hospitalization day	0.185	0.034	0.020
NIHSS	−1.219	0.097	0.018

**Table 5:** Logistic regression analysis between PNI and clinical parameters in patients with acute ischemic stroke.

Parameters	β	*p* Value
Infection	0.188	0.004
NIHSS	1.219	0.018
Atrial fibrillation	1.225	0.031
Mortality	5.083	0.016

## Data Availability

All data generated or analyzed during this study are included in this article. Further inquiries can be directed to the corresponding author.
